# Research on Carbide Characteristics and Their Influence on the Properties of Welding Joints for 2.25Cr1Mo0.25V Steel

**DOI:** 10.3390/ma14040891

**Published:** 2021-02-13

**Authors:** Qing Li, Guangxu Cheng, Mu Qin, Yafei Wang, Zaoxiao Zhang

**Affiliations:** School of Chemical Engineering and Technology, Xi’an Jiaotong University, Xi’an 710049, China; liqingl@stu.xjtu.edu.cn (Q.L.); qinmu.1990.0113@stu.xjtu.edu.cn (M.Q.); yafeiwang@mail.xjtu.edu.cn (Y.W.); zhangzx@xjtu.edu.cn (Z.Z.)

**Keywords:** 2.25Cr1Mo0.25V, hydrogen diffusion, carbides, microstructure

## Abstract

The carbide characteristics of 2.25Cr1Mo0.25V steel have an extremely important influence on the mechanical properties of welding joints. In addition, hydrogen resistance behavior is crucial for steel applied in hydrogenation reactors. The carbide morphology was observed by scanning electron microscopy (SEM) and the carbide microstructure was characterized by transmission electron microscopy (TEM). Tensile and impact tests were carried out and the influence of carbides on properties was studied. A hydrogen diffusion test was carried out, and the hydrogen brittleness resistance of welding metal and base metal was studied by tensile testing of hydrogenated samples to evaluate the influence of hydrogen on the mechanical properties. The research results show that the strength of the welding metal was slightly higher and the Charpy impact value was significantly lower compared to the base metal. The hydrogen embrittlement resistance of the welding metal was stronger than that of the base metal. The presence of more carbides and inclusions was the main cause of the decreased impact property and hydrogen brittleness resistance of the welding metal. These conclusions have certain reference value for designing and manufacturing hydrogenation reactors.

## 1. Introduction

Hydrogen reactors are necessary and very important equipment in the petrochemical and coal chemical industries. Material damage or failure can occur when the reactor operates under high temperature and high pressure and is in contact with hydrogen for a long period. Therefore, toughness and hydrogen resistance of the material and its welding joints are expected and have been a focus of researchers. Two typical materials for hydrogenation reactors are 2.25Cr1Mo and 2.25Cr1Mo0.25V steel. In the past few decades, a large amount of literature has become available on 2.25Cr1Mo steel, including its mechanical properties [1,2], microscopic properties [3,4], reheat cracking properties [5,6], and hydrogen environmental properties [7,8,9,10]. In recent years, 2.25Cr1Mo0.25V steel has been widely used in hydrogen reactors instead of the traditionally used 2.25Cr1Mo steel, due to its excellent hydrogen resistance, high-temperature strength, and other mechanical properties. However, the influence of welding and post-weld heat treatment (PWHT) on its impact properties and carbides is less studied.

The manufacturing requirements for 2.25Cr1Mo0.25V steel are higher than those for 2.25Cr1Mo material [11]. Generally, it is expected that the performance of the welding metal (WM) will be similar to or even better than that of the base metal (BM), but the actual results are not always satisfactory, since the welding thermal effect and impact toughness of the WM are usually lower than those of the BM. Kim et al. [12] simulated the post-welding heat treatment process and found that the impact energy of the material decreased significantly when the ferrite content increased with heat treatment. Song et al. [13] conducted a series of notch experiments under conditions of PWHT and tempering. They concluded that the Charpy impact toughness of the welding metal was worse and proposed a threshold value of impact energy greater than 54 J at −40 °C.

A large amount of second-phase carbide is characteristic of the 2.25Cr1Mo0.25V steel bainite phase [14]. Sakai’s study [15] showed that the addition of V element could improve the material’s high-temperature resistance to hydrogen brittleness. Sakai also reported that V-rich carbides are thought to be responsible for the fining and stabilizing of carbides, which improve resistance to hydrogen embrittlement and attack, respectively. Lee et al. [16] found that with increased V content, the captured hydrogen content in steel increases. When the V content is 0.2%, the steel shows the best resistance to hydrogen embrittlement. When the V content increases incrementally, the resistance to hydrogen embrittlement becomes worse, which may be related to the large number of undissolved carbides caused by excessive V. Song et al. [14] simulated the welding process and conducted impact tests on different areas of welded joints. The results revealed that the presence of pre-charged hydrogen caused a significant decrease in fracture toughness for both the BM and WM specimens. Moreover, Hui et al. [17] conducted slow tensile tests for different ferritic steels and pointed out that bainite was more susceptible to hydrogen embrittlement than pearlite due to its higher strength and microstructure characteristics. Shi et al. [17] pointed out that hydrogen-induced cracks began to grow along hard grain boundaries such as large martensite-austenite (MA) islands and bainitic ferrite. The second phase is the critical factor affecting the mechanical properties and hydrogen embrittlement resistance.

The heat treatment time and welding technique will cause carbides to change [18] and the effect of carbide on performance is nonnegligible. As for the current methods of designing hydrogenation reactors, uniform physical parameters are usually used to check the strength and structure [19]. The weaker parts of the overall structure determine the product performance after welding and heat treatment, and the properties of the WM are more representative of these parameters. Therefore, the present study aimed to analyze the role of the second phase in the performance difference between the BM and WM and provides a useful theoretical reference for improving the hydrogen environmental service performance of reactors.

## 2. Materials and Methods

**Figure 1:**
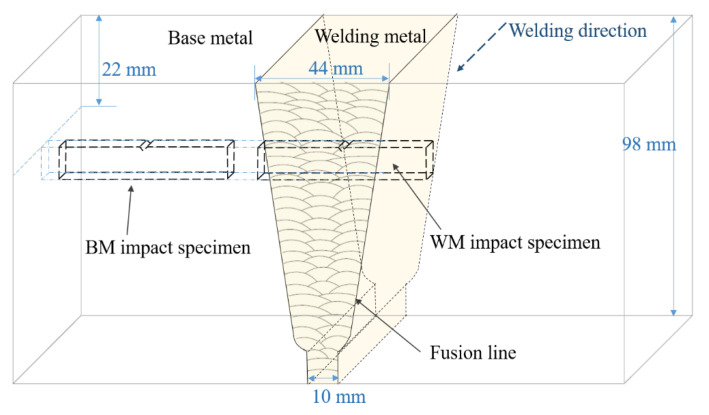
Diagram of welding test plate and sampling position of impact specimen.

The experiment used 2.25Cr1Mo0.25V, a kind of low-alloy high-strength steel. The steel plate was produced by WuSteel Ltd. (Wugang, China), and the quality conformed to ASME SA-542/SA-542M [20]. The size and shape of the welding test plate are shown in Figure 1, and the welding length was 800 mm. Based on the actual production procedure of the manufacturing factory, the plate was preheated at about 180 to 220 °C and welded by submerged arc welding (SAW) with US-521H as the filler metal and PF500 as the flux under 580 A current and 32 V voltage with multiple layers and passes. Then, the welding residual stress was released by post-welding heat treatment at 705 °C for 8 h. The whole welding process met API RP 934-A-2019 [21] standards. The thickness of the 2.25Cr1Mo0.25V steel plate was 98 mm, and a single V-groove was machined on one side of the plate. The whole joint was welded 79 times to fill the groove. The compositions of the WM and BM were analyzed, as listed in Table 1.

### 2.1. Mechanical Property Test

**Figure 2:**
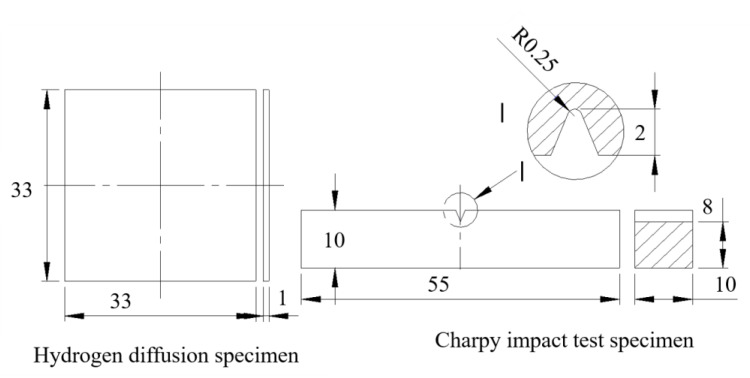
Dimensions of hydrogen-permeable specimen and impact specimen (mm).

The middle section of the welding test plate was selected for the experiment. A conventional tensile test and impact test on the BM and WM were carried out. These tests were performed at least three times in each state. The location of the cutting sample for the Charpy impact value is shown in Figure 1; the cutting locations of the tensile test sample and hydrogen-charged tensile test were the same as the Charpy specimens, and the sampling axial direction was parallel to the welding direction. The specimen size for the Charpy impact test is shown in Figure 2. A V-notch was adopted for impact specimens, following ISO 377-2017 [22]. Impact tests were carried out with a PSW750 impact tester and conducted under 6 different temperatures from 20 to −80 °C following ISO 148-1-2016 [23].

**Figure 3:**
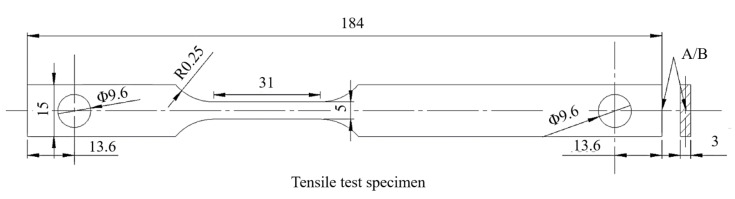
Dimensions of tensile specimen (mm).

Tensile specimens were rectangular sections with a diameter of 5 × 3 mm^2^ and a parallel section length of 30 mm; the tensile test is shown in Figure 3. To be consistent with the hydrogen-charged tensile specimen, a special asymmetric design was adopted for the tensile specimen. Tensile tests were conducted on an Instron 3382 universal testing machine (Instron corporation, Norwood, MA, USA) at 2 mm/min (6.67 × 10^−4^/s). The experimental process and sample met the standard of ISO 6892-1-2016 [24].

**Figure 4:**
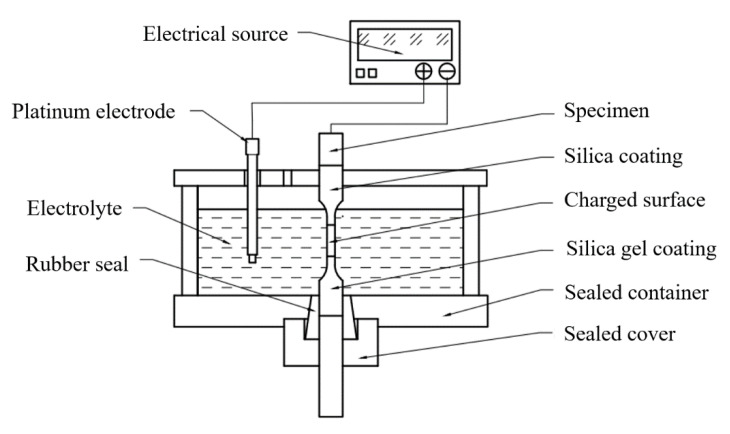
Schematic diagram of synchronous hydrogen charging tension.

As shown in Figure 4, to achieve the tensile test for loading with synchronous hydrogen charging, the specimen was placed in a sealed container that also acted as an electrolyzer. To put enough distance in the sealing part, the extension of the clamping end was designed to be on one side. This part of the tensile test did not use the extensometer for the addition of the electrolytic cell. Insulated silica gel was coated on both ends of the part of the specimen in the container, and a certain area was set aside in the middle to control the current density of hydrogen charging. The upper part of the specimen was connected with a wire to the negative pole of the constant current source, and the platinum electrode was connected to the positive pole of the constant current source to form an electrolytic cell. A 0.5 mol/L H_2_SO_4_ + 500 mg/L Na_4_P_2_O_7_ solution was used as the electrolyte. The cathodic current was kept at 128 mA (40 mA/cm^2^ for the working surface) to charge the specimen. To simulate the working condition of the material in a hydrogen environment, hydrogen charging was carried out for 2 h before the tensile test, because the literature [25] indicates that 2 h of hydrogen charging can cause enough hydrogen to penetrate into the material.

### 2.2. Hydrogen Electrochemical Permeation Tests

To measure hydrogen diffusion, specimens from the permeation test were cut from the test board by wire cutting to 33 × 33 mm^2^ size and 1 mm thickness. The specimens were polished with 100 # to 2000 # sandpaper, cleaned, and preserved. To prevent hydrogen-charged acid from corroding the specimens and promoting hydrogen oxidation on the anode surface, and to avoid hydrogen bonding to hydrogen on the anode surface, a metal nickel coating on the anode surface of the specimen was required. The formula of the nickel-plating solution was 250 g/L NiSO_4_ 6H_2_O + 45 g/L NiCl_2_ 6H_2_O + 40 g/L H_3_BO_3_. The current density of the plating was 2.8 mA/cm^2^, the plating time was 5 min, and the plating temperature was 55 °C. The coating on one side was sanded and the thickness of the specimen was controlled to 800 microns.

**Figure 5:**
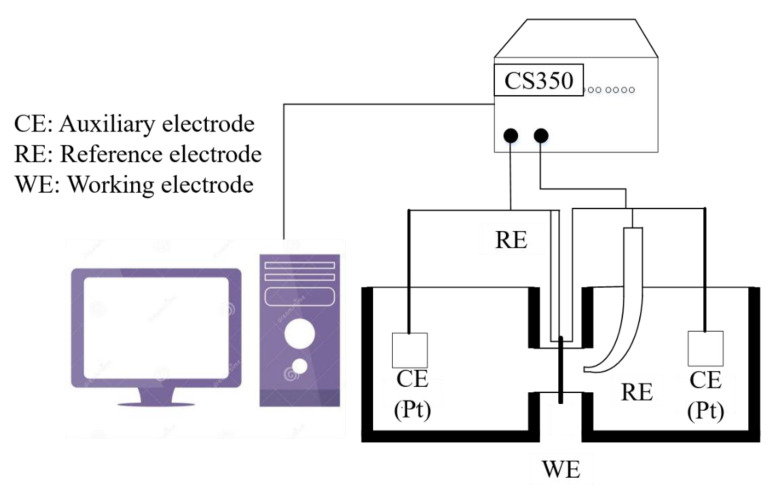
Hydrogen electrochemical permeation device.

Hydrogen electrochemical permeation tests were performed following the method developed by Devanathan and Stachurski [26]. As shown in Figure 5, the hydrogen seepage device of the Davanathan–Stachursky double electrolytic cell was used in the experiment. The device consists of a cathode hydrogen charging chamber on the left side and an anodic oxidation chamber on the right side. The thin specimen was sandwiched between the two cells and fixed by a fixture. NaOH solution of 0.2 mol/L was added to the right anodizing chamber and 0.5 mol/L H_2_SO_4_ + 500 mg/L Na_4_P_2_O_7_ solution was added to the left cathode charging chamber. Na_4_P_2_O_7_ was used as a poisoning agent to prevent hydrogen atoms from binding to hydrogen molecules on the surface of the hydrogen-charging side of the specimen and promote hydrogen permeation. During the test, the specimen was clamped between two electrolytic cells with the fixture. The exposed area of the specimen was 2.27 cm^2^. The main unit of the CS2350 electrochemical workstation was connected to the anodic oxidation chamber on the right side. The working electrode (WE) was connected to the specimen, the reference electrode (RE) was connected to the Hg|HgO electrode, the auxiliary electrode (CE) was connected to the platinum electrode, and the NaOH solution was poured into the anode chamber. A constant potential polarization of 300 mV was applied on the anode side. When the oxidation current was stable, 0.5 mol/L H_2_SO_4_ + 500 mg/L Na_4_P_2_O_7_ solution was added to the cathode hydrogen charging chamber, and the current in the cathode side was kept constant at 90.8 mA (40 mA/cm^2^). The unit was connected to the cathode hydrogen charging chamber on the left side. WE and RE were connected to the specimen, and CE was connected to the platinum electrode. At this time, the system began to record the oxidation current curve. When the current rose to a certain level, the oxidation current curve remained stable. After the test, the recorded oxidation current curve became the specimen hydrogen permeation curve.

For the hydrogenation permeation test, to satisfy Fick’s first and second laws [27], an equation was obtained through variable separation and approximation in the specific experiment [28]:

(1) $$\frac{i(t)}{i_{\infty }}=\frac{J(t)}{J_{\infty }}=1-2e^{-\pi^{2}\tau }$$

where $\tau =\frac{Dt}{d^{2}}$; i(t) is the current at time t(s), J(t) is the diffusion flux of hydrogen at time t, $i_{\infty }$ (is the steady current, $J_{\infty }$ is the steady diffusion flux of hydrogen, *D* is the hydrogen diffusion coefficient, and d is the specimen thickness (mm).

Then, we have these relations [29]:

(2) $$J_{\infty }=\frac{i_{\infty }}{AF}$$

(3) $$D=\frac{d^{2}}{15.3t_{b}}$$

where $t_{b}$ is the time when the anode current density begins to rise (s), *A* is the diffusion area (cm^2^), and *F* is the Faraday constant.

Oriani’s formula approximates the hydrogen trap density [30]:

(4) $$c_{f}=\frac{i_{\infty }d}{FD}$$

(5) $$N_{T}=\frac{c_{f}}{3}(\frac{D_{L}}{D}-1)$$

where $c_{f}$ is the hydrogen concentration (mol cm^−3^), $N_{T}$ is the number of hydrogen traps (cm^−3^), and $D_{L}$ is the diffusion coefficient of hydrogen in the lattice (1.28 × 10^−4^ cm^2^ s^−1^) [31].

Dislocations and second phases in the material are often considered hydrogen traps during hydrogen diffusion [32,33,34]. Many studies have shown that hydrogen traps in metal materials can be divided into reversible and irreversible hydrogen traps [35,36]. During the experiment, hydrogen diffused into the material was first captured by various irreversible hydrogen traps and filled with various reversible hydrogen traps, and finally diffused to the other side, gaining electrons and leaving the metal. Before the second hydrogen diffusion section, using an electromotive force, the hydrogen are released from the reversible hydrogen trap of the metal. In the second hydrogen diffusion, only the reversible hydrogen trap was filled and eventually diffused. Therefore, in principle, the number of hydrogen traps obtained from the first hydrogen diffusion is the sum of the reversible and irreversible hydrogen traps, the number of traps obtained from the second hydrogen diffusion is only the number of reversible hydrogen traps, and the encircling area of the first and second hydrogen filling curves is the amount of hydrogen absorbed by the irreversible hydrogen trap.

### 2.3. Morphology Observation

For the metallographic structure observation by SEM, specimens with dimensions of 10 mm × 10 mm × 5 mm were cut from the plate. They were ground gradually with 100 # to 2000 # sandpaper, polished with 1 µm diamond polishing agent, and cleaned. Fracture specimens were made by wire cutting near the fracture surface of the tensile specimen, with a notch about 10 mm away from the fracture surface. Electron backscatter diffraction (EBSD) samples were further ion-thinned based on SEM samples to remove the stress layer on the surface. The morphology was observed using the MAIA3 SEM (Tescan, Brno, Czech Republic), which has the function of energy dispersive x-ray spectroscopy (EDX). Transmission electron microscopy (TEM) with functions of selected area electron diffraction (SAED) and electronic energy loss spectrum (EELS) was used to characterize the specimen morphology with a JEM-2100 TEM (Akishima, Tokyo, Japan). Observations were conducted to characterize matrix phases and carbides. Thin foils with dimensions of 10 mm × 10 mm × 0.3 mm were cut from the welding plate, ground to 70 µm (thickness) with sandpaper, and twin-jet electropolished in an electrolyte comprising 5% perchloric acid and 95% methanol. The polishing current was controlled at 40 mA.

### 2.4. Thermodynamic Property Calculation

According to the principles of thermodynamics, the general conditions for the system to reach equilibrium at constant temperature and pressure are as follows [37]:

1. The total Gibbs free energy (*G*) of the system reaches the minimum value.
2. The chemical formula of component “*i*” in the system is equal.

For each phase [38],

(6) $$G_{m}=\sum_{i}X_{i}G_{i}^{0}+RT\sum_{i}X_{i}\ln X_{i}+\sum_{i}\sum_{j}X_{i}X_{j}\sum_{\upsilon }\Omega_{\upsilon }{\left(X_{i}-X_{j}\right)}^{\upsilon }$$

where $G_{m}$ is Gibbs free energy, $X_{i}$ is the mole fraction of component *i*, $G_{i}^{0}$ is the standard Gibbs free energy of component *i*, *R* is the perfect gas constant, *T* is the absolute temperature, and $\Omega_{\upsilon }$ is a combinatorial number.

In this paper, the thermodynamic properties module of JMatPro software (Version 9.0, Sente Software Ltd., Guildford, UK) [39] was used to calculate the temperature-phase composition diagram for the temperature range of 400 to 1600 °C, with calculations made every 10 °C.

## 3. Results and Discussion

### 3.1. Comparison of WM and BM Mechanical Properties

**Figure 6:**
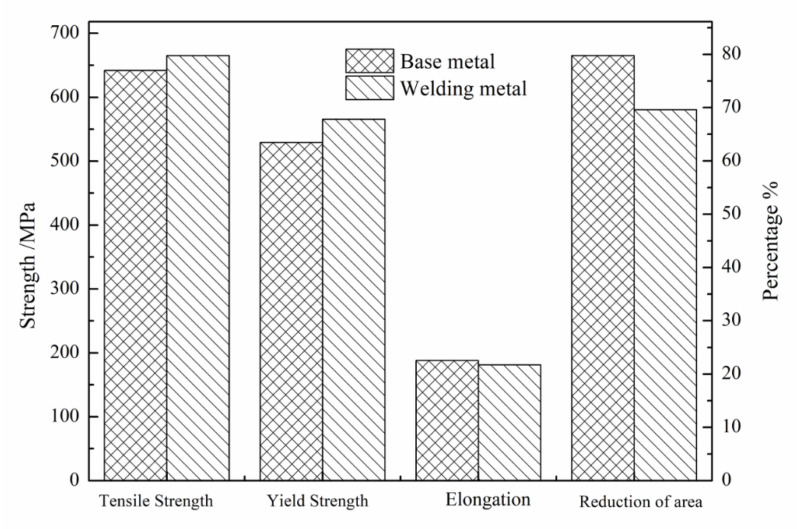
Tensile properties of BM and WM.

The tensile test results of the BM and WM under selected welding and heat treatment processes are shown in Figure 6. The yield strength and tensile strength of the WM were slightly higher than those of the BM. The elongation after fracture of both was above 20%, indicating excellent plasticity.

**Figure 7:**
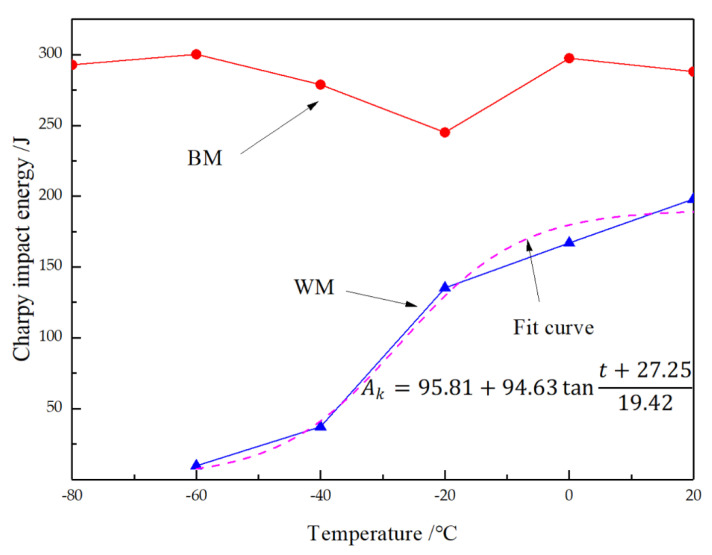
Comparison of Charpy impact energy under different temperatures for BM and WM.

The impact energy is a critically important parameter for describing toughness in actual engineering design. A comparison of the impact test results for the BM and WM is shown in Figure 7; it can be seen that the two had significantly different toughness. This is manifested in two aspects. First, for BM, the impact test results at room temperature showed that the impact absorption energy was up to 250 J at −20 °C, but for the WM, the impact absorption energy was only 153 J at −20 °C, which is half that amount. The requirement [21] is that tensile stress should be less than 760 MPa and the impact energy should be higher than 55 J at −29 °C; such data are considered acceptable. However, significant differences between the BM and WM may lead to discontinuity in the performance of the overall structure and more problems during operation. Second, as seen in Figure 7, with decreased temperature, the impact energy of the BM remained almost unchanged, at higher values. This indicates that the ductile–brittle transition temperature of the BM was lower than −80 °C, and it can serve well in both conventional and cold regions. However, the impact absorption energy of the WM was much lower than that of the BM under all five temperatures. The impact absorption energy declined remarkably as the temperature dropped. In the temperature range tested, the impact energy-temperature curve of the WM shows a transition zone from ductile to brittle. According to the fitting curve of the impact energy curve of the WM (Boltzmann function shown in Figure 7), the ductile and brittle transition temperature was determined to be −27.2 °C according to 50% of the upper and lower platform interval of the impact energy. Such a temperature can be reached in winter in many areas of northern China, so it may be dangerous for hydrogenation reactors exposed to the outside in normal service.

**Figure 8:**
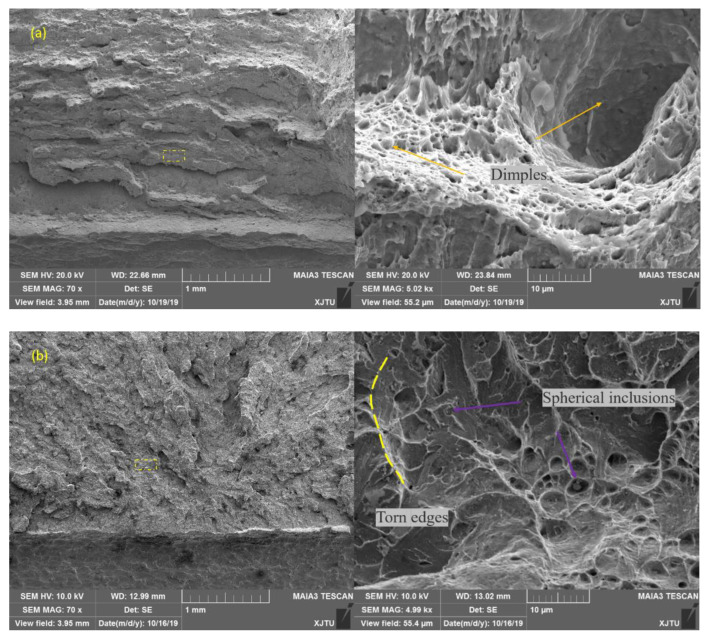
Comparison of fracture morphology of impact specimen for (**a**) BM and (**b**) WM by SEM (scanning electron microscopy) (−20 °C).

**Figure 9:**
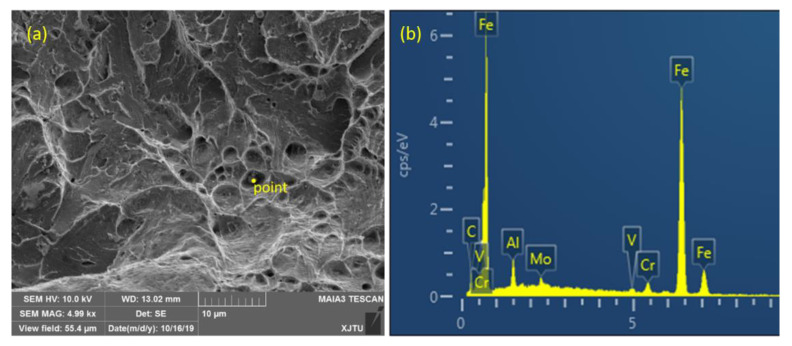
Analysis of inclusion composition on fracture of WM for (**a**) position and (**b**) content.

In Figure 8 and Figure 9, further analysis of the fracture surface showed that there were many dimples in the initial fracture area of the impact fracture of the BM, which can absorb a lot of impact energy. However, the fracture of the WM was a quasi-cleavage area mixed with a dimpling and de-factoring surface, with a low capacity for absorbing impact energy. Further analysis showed that the second phase of the granular form was widely distributed on the de-factoring platform at the bottom of the dimpling of the WM. The chemical composition of the carbide and inclusions precipitated in the matrix were analyzed by EDX, and the results show that the particles were carbides with multiple alloying elements.

The main reason why the impact energy of the WM was half of the BM was because of carbides and other second phases. A large amount of second-phase carbides in the WM destroys the continuity of the matrix. When subjected to an instantaneous load, the phase interfaces are prone to increased crack initiation and decreased impact performance.

### 3.2. Influence of Hydrogen on Tensile Strength and Fracturing

**Figure 10:**
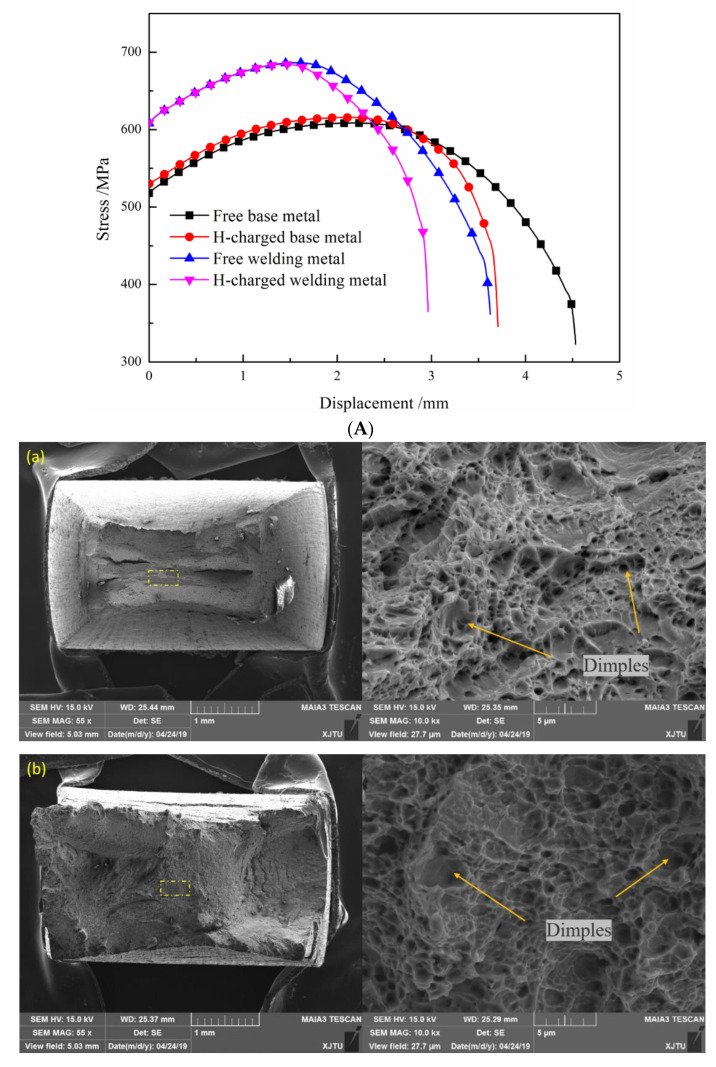

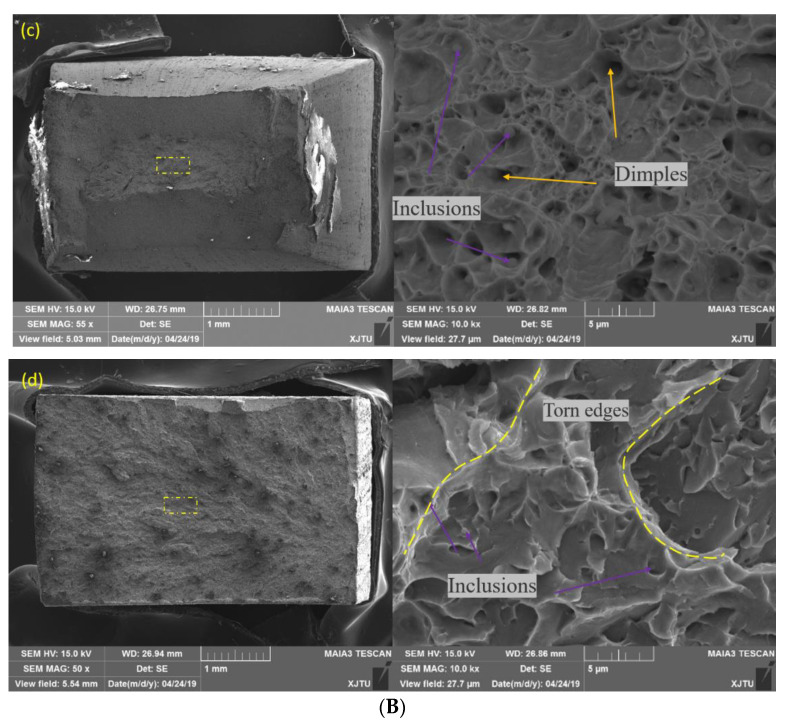
Hydrogen-charged tensile properties of BM and WM. (**A**) Tensile displacement curve. (**B**) Morphology of hydrogen-charged slow tensile fracture: (**a**) free-BM; (**b**) charged BM; (**c**) free-WM; (**d**) charged WM.

The hydrogen-charged tensile test and fracture results of the BM and WM are shown in Figure 10. Since the experimental device cannot hold the extensometer, the abscissa represents the stretching displacement of the clamp. In the tensile test without hydrogen charging, the yield strength and tensile strength are higher, and the displacement at the fracture of the WM is significantly lower compared to the BM, which is consistent with the experimental results of the tensile test with extensometer in Section 3.1. Both the BM and WM showed a decrease in plasticity after pre-charging and synchronous charging. Both metals reached the necking state in advance after hydrogen charging. The failure occurred quickly after necking, and the final fracture displacement was significantly smaller than that of the sample without hydrogen charging. For hydrogen charging samples, the tensile fracture displacement decreased by 21.25% for the BM and 18.2% for the WM. The gap between the two values is not big. However, the effect of hydrogen on the BM and WM can be seen more directly through the change of reduction of area (RA) of fracture before and after hydrogen charging. Table 2 lists the statistics of changes in the RA of the BM and WM. After hydrogen charging, the RA of the BM decreased by 34.47%, while that of the WM decreased by 84.16%. This indicates that hydrogen has a more significant effect on the performance of WM than BM. This phenomenon of ductile loss after hydrogen charging is in agreement with the previous study [40,41,42].

The same conclusion can be drawn from the tensile fracture, as shown in Figure 10. For the BM, dimple size became shallow after hydrogen charging; that is, hydrogen reduced the plasticity. However, the tensile fracture of the BM had plastic fracture morphology before and after hydrogen charging. On the contrary, the tensile fracture morphology of the WM before and after hydrogen charging changed greatly. Before hydrogen charging, the WM fracture morphology was plastic, with large dimples in the middle and spherical inclusions at the bottom; after charging, it changed to brittle, and the core was small cleavage platforms and tearing edges. The resistance to hydrogen was the same as the impact property. More precipitates resulted in a greater reduction of the plasticity of the WM than the BM after hydrogen charging.

### 3.3. Analysis of BM and WM Carbide Characteristics

Influenced by the welding and post-treatment thermal cycle, the change of the BM microstructure was mainly manifested as carbide precipitation, as the temperature was below the phase transition temperature. Coarse columnar crystals originally formed in the cooling process of the WM were gradually refined in the subsequent welding process, and precipitated out more carbides. Meanwhile, there were spherical oxide inclusions due to the invasion of ambient oxygen.

**Figure 11:**
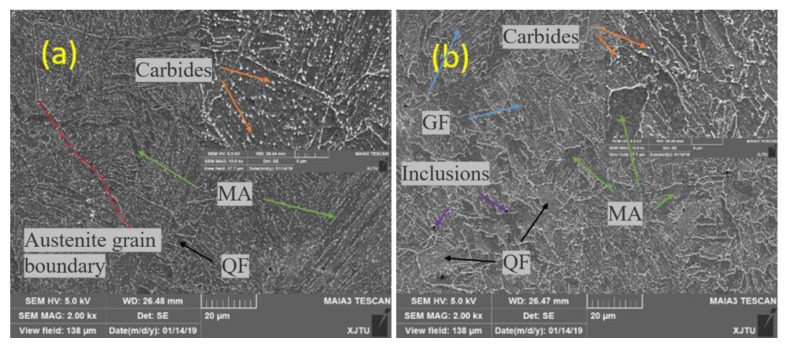
SEM observation: (**a**) BM and (**b**) WM.

The SEM observation results are shown in Figure 11. The SEM images of the BM show that the grain boundary of the original austenite (dashed red line) is obvious and most of the inner part was lamellar ferrite. The martensite-austenitic (MA) islands of granular precipitation (the green arrows) were almost uniformly distributed, which is still a typical granular bainite (GF) structure (blue arrows). A small amount of quasi-polygonal ferrite (QF; black arrows) was also seen. These results are similar to Jiang’s [43]. Compared with the BM, the WM presented a great difference. The QF increased significantly, and the precipitation of MA islands became more irregular. The MA islands in the WM were larger than that in the BM, and there were large inclusion particles. The carbide precipitation (orange arrows) in the BM was more uniform, but it was not uniform in the WM which showed local enrichment.

**Figure 12:**
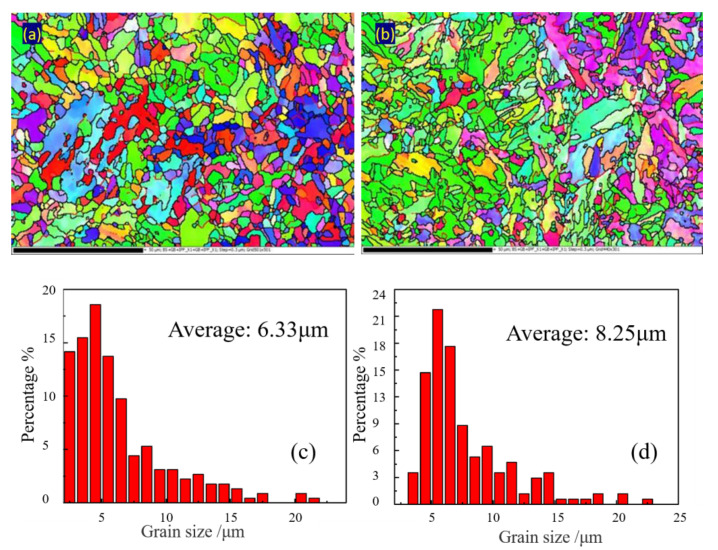
Effective grain size statistics: (**a**,**c**) BM and (**b**,**d**) WM.

EBSD was used to analyze the size of the microstructure, as shown in Figure 12. Statistics of sizes showed that BM had a smaller grain size than the WM. The average grain size of the BM and WM was 6.33 µm and 8.25 µm, respectively. To further analyze the difference in microstructure of these two metals, the metal microstructure and particles were further identified and observed by TEM.

Figure 11 shows the TEM micrographs of the BM and WM. The matrix structure of the BM (Figure 11a) is a GF structure with an obvious lamellar contour and MA islands. The carbon-rich MA islands and granular carbides are distributed in the bands with some regularity. There is no obvious deformation trend in the MA islands, and there is a flocculent dislocation structure around the MA islands and carbides. Compared with the BM, in addition to GF and MA islands, the matrix structure of the WM has a more complex occurrence of radially shaped intragranular acicular ferrite (IAF) surrounding the inclusions. At lower temperature, a small amount of austenite in the tip clearance gradually accumulated carbon and converted it into MA, and the large spherical inclusion in the WM provides the core for the formation of IAF. The increased dislocation clusters around the MA islands indicates that the crystal defects in the structure were increased, which may be the reason why the WM was slightly stronger than the BM.

There were a lot of spherical inclusions in the welds. The size of inclusions formed by welding was about 500 to 1000 nm because the WM is inevitably invaded by ambient oxygen and contains many spherical oxide particles. More MA islands mean more brittle second phases, which have a certain destructive effect on the toughness of the material. These inclusion particles are the initiation source of the impact cleavage fracture, and even cause a decrease in the hydrogen-induced cracking (HIC) performance of the material [44].

Widely distributed carbides in metal are of concern. The precipitation of large carbide particles reduces the utilization rate of micro-alloy elements in materials; small-sized carbides can pin grain boundaries to refine grains, and smaller dispersed carbides have a strengthening effect on the material.

**Figure 13:**
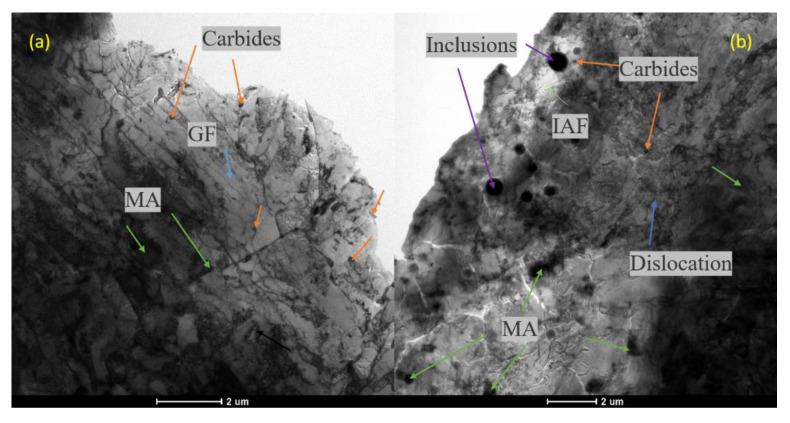

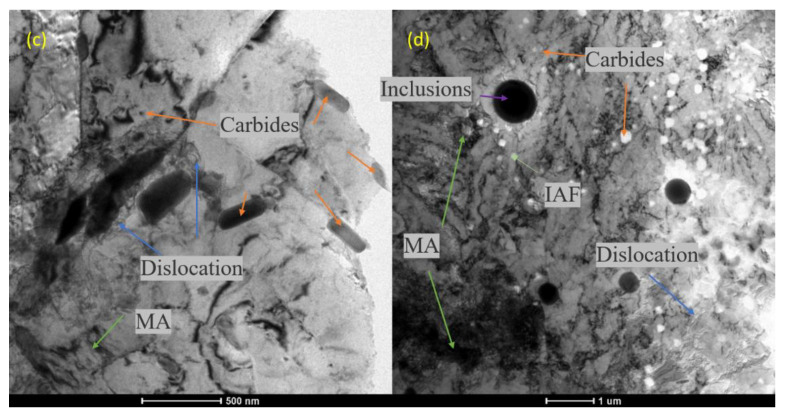
TEM diagrams: (**a**) slab matrix structure, martensite-austenitic (M-A) islands in BM; (**b**) matrix structure, MA islands in WM; (**c**) carbides; (**d**) spherical inclusions in WM.

**Figure 14:**
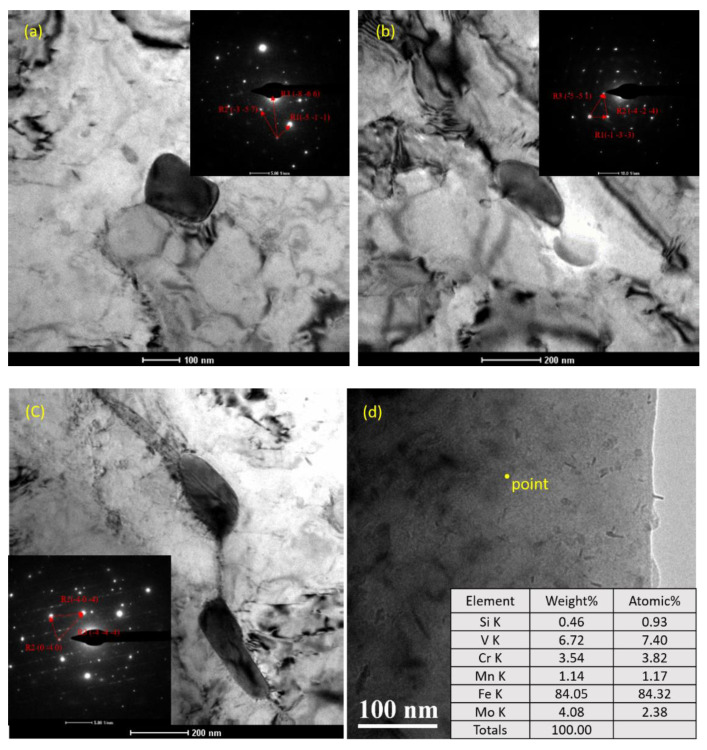
Carbide composition calibration in BM and WM: (**a**) M_23_C_6_; (**b**) M_2_C; (**c**) M_6_C; (**d**) MC.

It can be seen from Figure 13 that carbides were widely distributed in the BM and WM, and the size of large particles was about 200 to 300 nm. Figure 14 was used to calibrate the main components of carbide in the BM by the diffraction spot in SAED analysis. The results show that the carbide composition in the sections shown in Figure 14a–c was M_23_C_6_, M_2_C, and M_6_C, respectively. In addition, many small granular and circular flake particles can be seen in the vicinity of the dislocation by magnification. For the finer carbide particles in Figure 14d, the diffraction spot could not be accurately carried out, so EELS was used to roughly calibrate the composition, and the result showed that the carbon and vanadium content in these carbide particles was higher than the average level of the material; it was believed that there was an enrichment of V carbide. Compared with what is reported in the literature [45], this kind of granular carbide may be VC or V_4_C_3_.

**Figure 15:**
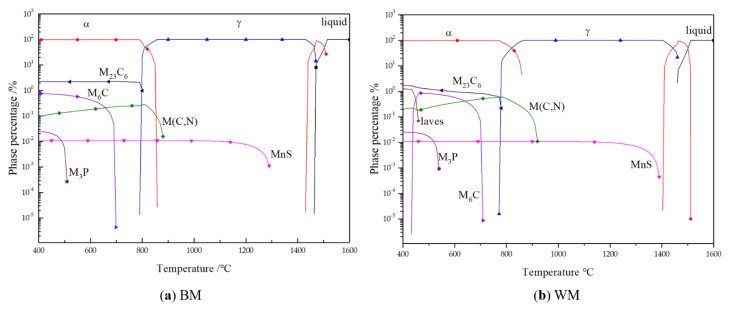
Calculation of phase precipitation in equilibrium cooling process: (**a**) BM; (**b**) WM.

Because the products of carbide observed by TEM can only prove the existence of a certain carbide inside the metal, but not the formation time and the whole composition of carbide, JMatPro software was used to simulate the balanced cooling process of BM and WM. The chemical composition of the welded structure of BM and WM was determined, and the carbide precipitation during the cooling process of welding was simulated. The result is shown in Figure 15.

Figure 15 shows the phase composition of the BM and WM cooled from 1600 to 400 °C in equilibrium. The simulation results show that the composition of carbides in the BM and WM was similar, which was consistent with the result of the TEM calibration. During the equilibrium cooling process, the MC phase precipitated out before the ferrite transformation and existed near the original austenite grain boundary in the matrix. The M_23_C_6_ phase precipitated out at the ferrite transition stage and existed at the ferrite lath boundary. Because of the high inclusion element content, MnS and M_3_P phases were generated, in which MnS was precipitated at the grain boundary of pro-ferrite, and the growth time was long enough for the particles to be large. M_3_P precipitated out at a low temperature. Higher Mn content leads to a higher precipitation temperature of MnS and a larger precipitation phase size at room temperature.

Carbides of different sizes precipitate at different temperatures, and they play different roles in steel. The carbides shown in the figure at about 200 nm were M_23_C_6_ and M_6_C precipitated at higher temperatures when the material was cooled. The main function of this carbide is to pin grain boundaries and prevent austenite grain growth. However, when the temperature is high, it is easy to coarsen for a long time, and the material containing this carbide cannot keep the austenite grain for a long time at a high temperature. Another kind of carbide with a size less than 30 nm was mainly composed of V(C, N), which was precipitated from the austenite-ferrite transformation process and supersaturated solid solution alloy elements cooled to the ferrite region. The volume fraction of this size carbide in the matrix was very small, but the strengthening effect on the matrix was very obvious. Some of these fine-needle carbides also exist in dislocation clusters. The MC diffused through the dislocation grew very slowly, and remained fine after tempering. The interaction between these uniformly dispersed fine precipitates and the dislocation could effectively enhance the strength of the tissue. However, some studies [46] have pointed out that dispersed distribution formed precipitated reinforced carbides such as VC, which would cause it to be prone to reheat cracks.

Therefore, compared with the base metal, the weld had more MA islands, dislocations, and carbides, and more inclusions were introduced during the formation process. Altogether, these factors led to significant differences in the mechanical properties of the WM.

### 3.4. Hydrogen Diffusivity and Hydrogen Trap

**Figure 16:**
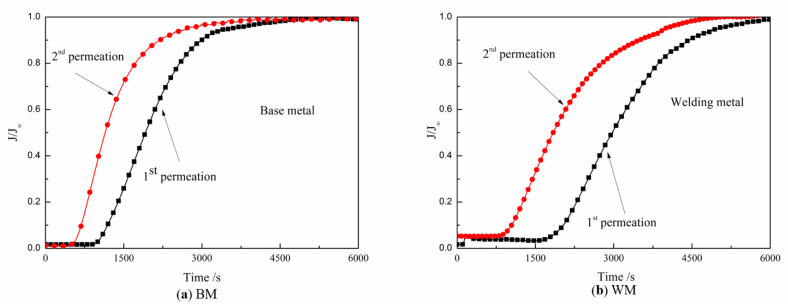
Hydrogen diffusion curve of sample BM and WM at 20 °C: (**a**) BM and (**b**) WM.

**Figure 17:**
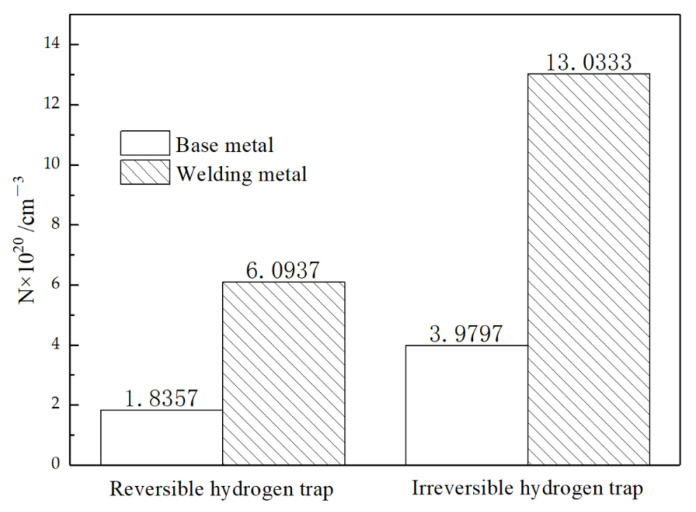
Comparison of hydrogen trap number between BM and WM.

Figure 16 shows the curves of the BM and WM for hydrogen charging two times. After charging, the current density started to increase for a while, and then the growth rate slowed down and reached a stable value. The first charging process took longer than the second infiltration to achieve equilibrium. The experimental results show that the BM had far less penetration time than the WM in both the first and second hydrogen charging. The penetration time of the BM in the first and second hydrogen diffusions was 44.2 and 51.92% less than that of the WM, respectively. Comparing the hydrogen diffusion curves of the BM and WM, it can be seen that hydrogen quickly passed through the metal and reached the equilibrium state in the BM. It can be intuitively seen from the area enclosed by the two curves in Figure 16 that there were more irreversible hydrogen traps in the weld. The diffusion parameters were calculated using Equations (1)–(5), and the results are shown in Table 3 and Figure 17. Both irreversible and reversible hydrogen traps in the WM were significantly higher than those in the BM.

It is obvious from Figure 17 that there was a huge difference in the number of hydrogen traps between the BM and WM. It is generally accepted that dislocation is a reversible hydrogen trap. The microstructure study of the BM and WM shows that there were many dislocation cells in the WM, and the flocculent dislocation group exists in all parts of the matrix. Studies on the microstructure of the BM and the WM showed many dislocation cells in the WM and flocculent dislocation groups in all parts of the matrix, although the grain of the BM was slightly smaller than that of the WM. The rule that the number of reversible hydrogen traps was higher than that in the BM was also consistent with common sense.

In contrast to reversible hydrogen traps, the main existence mode of irreversible hydrogen traps in the materials is the phase interface of grain boundaries, MA islands, carbides, and inclusions [47]. From the microstructure, it can be seen that there were more carbides, more MA islands, and many inclusions in the WM. These second phases act as hydrogen traps and capture more hydrogen, leading to increased local hydrogen content in the metal. The bainite tissue itself was a fast channel for hydrogen [16], and the austenite was a relative binding point in the process of hydrogen diffusion. In the process of welding, the WM cools down rapidly, and ferrite transformation occurs before the austenite grains have grown into a complex matrix structure, so there were more MA islands in the WM at the same volume. It has been shown that the morphology of MA islands can significantly affect the hydrogen embrittlement resistance of the material [48]. Compared with the granular MA islands in the BM, the morphology of the MA islands in the WM was mostly blocky. For WM containing massive MA islands, the austenite in the MA begins to transform into martensite during the tensile process, resulting in the initiation of hydrogen-induced cracking (HIC) near the outer MA surface (due to local high hydrogen concentration). Once the main crack is formed, the stress concentrates in front of the crack tip. The crack promotes the localized transformation of the blocky MA and leads to more microcrack initiation. With repeated initiation and the continuous connection of cracks, the cleavage platform is finally formed, which causes brittle fracture of the material.

It has been pointed out in some literature [49] that most of the hydrogen in V-bearing steel is trapped by VC precipitation. However, the solubility of V in ferrite is 0.14 wt.%. An excessive concentration of V will form many V-rich carbides and granular V6C5 precipitates in the process of the thermal cycle. The V concentration of the WM reached 0.42 wt.%, as shown in Table 1, which is different from the optimal value given in [14]. These carbides absorb a large amount of hydrogen as hydrogen traps, inducing local brittle fracture and increasing the sensitivity of hydrogen embrittlement.

## 4. Conclusions

1. Tensile and impact tests were carried out on 2.25Cr1Mo0.25V steel and welding metal. The strength of the WM was slightly higher than the BM, but the toughness of the WM decreased mainly in two aspects: a reduction of fracture area in the tensile specimen and, more important, the impact energy. The impact performance of the WM was significantly lower than that of the BM, and the impact energy decreased obviously with decreased temperature. The ductility–brittleness transition temperature of the WM was −27.2 °C, so application in a low-temperature environment should be carried out with caution.
2. The synchronous hydrogen charging experiment results demonstrate that the BM appears to have stronger hydrogen embrittlement resistance than the WM. Before and after hydrogen charging, the BM showed a plastic fracture, and the dimples became shallower after hydrogen charging, with a hydrogen-induced section reduction of 34.47%. After hydrogen charging, the section reduction of the WM decreased by 84.16%, and the fracture changed from ductile to brittle. More precipitates and inclusions in the WM contributed to the lower performance of the WM than the BM.
3. The carbide characteristics were extensively studied by TEM. The microstructures of the BM and WM were observed. The matrix structure of the BM was uniform with fine GF and MA islands, and the WM was made up of QF, IAF, GF, and MA islands. There were more dislocations, inclusions, and carbides in the WM. The phases of M_23_C_6_, M_2_C, and M_6_C precipitated out and existed in both the BM and WM. Another kind of carbide with a size less than 30 nm was mainly composed of V(C, N). The presence of more carbides and inclusions was the reason why the WM had lower impact properties and hydrogen brittleness resistance than the BM.
4. The hydrogen permeation test showed that the apparent diffusion coefficient values were 4.60177 × 10^−7^ cm^2^ s^−1^ for BM and 2.56784 × 10^−7^ cm^2^ s^−1^ for WM, and there were more reversible (dislocation) and irreversible (carbide) hydrogen traps in the WM. Hydrogen enrichment in the WM would occur under the same hydrogen concentration gradient and the WM had a higher sensitivity to hydrogen.

## Figures and Tables

**Table 1:** Chemical composition of 2.25Cr-1Mo-0.25V steel (base metal, BM) and welding metal (WM) (wt.%).

Material	C	Si	Mn	S	P	Cr	Mo	V	Ni	Cu
BM	0.15	0.14	0.35	0.004	0.004	2.41	1.07	0.21	0.12	0.14
WM	0.12	0.22	1.07	0.004	0.004	2.45	1.03	0.42	0.03	0.11

**Table 2:** Section shrinkage of BM and WM in hydrogen charging tensile test.

Area and Percentage	BM		WM	
Original section area (mm^2^)	15		15	
Minimum cross-sectional area after tensile (mm^2^)	3.243	7.255	5.788	13.541
Reduction of section (%)	78.38	51.63	61.41	9.73
Hydrogen induced reduction (%)	34.47		84.16	

**Table 3:** Hydrogen diffusion data.

Material	Hydrogen Diffusion Time	*t*_b_ (s)	J_∞_ (10^−11^ mol cm^−2^ s^−1^)	D_eff_ (10^−7^ cm^2^ s^−1^)	c_f_ (10^−5^ mol cm^−3^)	$N_{T}\;(\times \;10^{20}\;{\mathrm{cm}}^{-3})$
BM	1st	909	2.64879	4.60177	1.0453	5.8154
	2nd	489	3.35123	8.55421	0.7114	2.1227
WM	1st	1629	2.79843	2.56784	1.9154	19.127
	2nd	1017	2.21652	4.11308	0.9786	6.0937

## Data Availability

The data used to support the findings of this study are available from the corresponding author upon request.
